# Analyses of CNS Response to Osimertinib in Patients with T790M-Positive Advanced NSCLC from ASTRIS Korean Subset, Open-Label Real-World Study

**DOI:** 10.3390/cancers13153681

**Published:** 2021-07-22

**Authors:** Beung-Chul Ahn, Jee Hung Kim, Kyoung-Ho Pyo, Sun Min Lim, Min Hee Hong, Hye Ryun Kim, Byoung Chul Cho

**Affiliations:** 1 Yonsei Cancer Center, Division of Medical Oncology, Department of Internal Medicine, Yonsei University College of Medicine, Seoul 03722, Korea; abcduke@yuhs.ac (B.-C.A.); PKHPSH@yuhs.ac (K.-H.P.); Limlove2008@yuhs.ac (S.M.L.); CBC1971@yuhs.ac (B.C.C.); 2 Division of Medical Oncology, Department of Internal Medicine, Gangnam Severance Hospital, Yonsei University College of Medicine, Seoul 06273, Korea; OK8504@yuhs.ac

**Keywords:** non-small-cell lung cancer, epidermal growth factor receptor, osimertinib, intracranial response

## Abstract

**Simple Summary:**

Patients with epidermal growth factor receptor (EGFR) mutation-positive non-small-cell lung cancer can have central nervous system (CNS) metastases during their disease course. A high unmet medical need exists especially for patients with T790M-positive NSCLC whose disease progressed after first-line EGFR-TKI. Osimertinib is a third-generation EGFR-TKI with selective activity for both sensitizing and EGFR T790M mutations and has improved CNS activity over first- and second-generation EGFR TKIs and chemotherapies. This study confirmed the clinical activity and CNS efficacy of osimertinib in an unselected real-world population.

**Abstract:**

Up to 40% of patients with epidermal growth factor receptor (EGFR) mutation-positive non-small-cell lung cancer (NSCLC) may develop central nervous system (CNS) metastases throughout their disease. Moreover, the first- and second-generation EGFR-tyrosine kinase inhibitors have limited efficacy because of their poor blood–brain barrier permeability. Therefore, we conducted preplanned analyses of ASTRIS, a clinical study of the third-generation EGFR-TKI osimertinib to demonstrate its potential role in intracranial response efficacies. We retrospectively examined 89 NSCLC patients with brain evaluation who were not amenable to curative surgery or radiotherapy and received osimertinib upon confirmation of the presence of the T790M mutation. We collected the information regarding patients’ baseline characteristics, baseline intracranial status, including leptomeningeal metastases (LM), and intracranial responses measured by Response Evaluation Criteria in Solid Tumors version 1.1, using independent central review. The median age was 60 years, and 69.7% of the patients were female. Sixty-five patients (73.0%) had brain metastases (BM) at baseline and nineteen patients (23.5%) had additional LM. Among patients with brain metastases, 24 (36.9%) had ≥1 measurable brain metastases and 16 were evaluated for the objective response. In the CNS evaluable for response set, the intracranial objective response rate (cORR) and disease control rate (cDCR) were 62.5% (95% confidence interval (CI), 38.3–82.6%) and 93.8% (95% CI, 74.3–99.3%), respectively. The median intracranial progression-free survival (cPFS) was 13.0 (95% CI, 7.21–18.8) months, including patients with measurable and non-measurable BM or LM. Our cORR, cDCR, and cPFS were comparable to those observed in previous clinical trials. The outcome of this study helps to demonstrate the potential role of intracranial efficacies of osimertinib 80 mg administration in T790M-positive advanced NSCLC with/without BM or LM.

## 1. Introduction

Central nervous system (CNS) metastases are one of the most lethal complications of epidermal growth factor receptor (EGFR) mutation-positive non-small-cell lung cancer (NSCLC). These metastases cause neurological dysfunction and cognitive impairment, which negatively affect the patient’s quality of life and survival [1]. Treating NSCLC metastases in the CNS with EGFR-tyrosine kinase inhibitors (TKIs) reduces CNS tumor progression more than cytotoxic chemotherapies and radiation therapies [2]. However, the activity of EGFR-TKIs is suboptimal for CNS cancer treatment, as EGFR-TKIs do not readily cross the blood–brain barrier (BBB). The CNS is considered a sanctuary for metastases, and many patients experience progressively worse symptoms due to CNS metastases [3].

Approximately 25% of patients with NSCLC have CNS metastases at the time of diagnosis, and 50% of the patients develop it throughout the disease, even after EGFR-TKI treatment [4]. Therefore, the treatment and prevention of CNS metastases is an important clinical goal of managing EGFR-mutant NSCLC.

Osimertinib is a third-generation EGFR-TKI with selective activity for both sensitizing and EGFR T790M resistance mutations. Preclinical studies showed that osimertinib had higher BBB permeability than gefitinib or erlotinib [5]. Furthermore, osimertinib has improved CNS activity over first- and second-generation EGFR TKIs and chemotherapies in patients with previously treated EGFR T790M-positive NSCLC and treatment-naïve EGFR-sensitizing mutation-positive NSCLC in phase III prospective trials [6,7].

The clinical activities and safety profiles of osimertinib were evaluated in ASTRIS trials (ClinicalTrials.gov identifier: NCT02474355) including more than 450 patients enrolled in Korea. This was a preplanned, exploratory analysis of CNS activity of osimertinib in the Korean subgroup of the ASTRIS trial to evaluate the efficacy and safety of 80 mg orally, once daily osimertinib. The study confirmed the clinical activity and a favorable safety profile of osimertinib in a real-world population [8]. Further, we provide preplanned analyses of osimertinib on the CNS metastases in a real-world cohort by blinded independent central review (BICR).

## 2. Materials and Methods

### 2.1. Study Overview

Patients were enrolled from 31 institutes across Korea, the details of which were published previously [9]. Patients with EGFR T790M mutation-positive NSCLC who had previously been treated with at least one EGFR-TKI, and for whom the disease had progressed, were invited to participate in the ASTRIS trial of osimertinib. Eligible patients continued to receive osimertinib at the investigator’s discretion until disease progression or until they experienced unmanageable drug-related toxicity.

### 2.2. Participants

All patients should have progressed on treatment with prior EGFR-TKI and with confirmation of the presence of the EGFR T790M mutation and been enrolled in the ASTRIS trial. Brain imaging by magnetic resonance imaging (MRI) or computed tomography (CT) was optional in the ASTRIS trial; therefore, only patients with baseline brain imaging (from within 3 months to enrollment date) were included in this ASTRIS CNS analysis study. All submitted brain scans were assessed by independent radiologists (BICR).

We divided the patients into three groups according to their initial assessment: (1) patients without brain metastases, (2) CNS evaluable for response set (cEFR), which included patients with one or more measurable CNS lesions, and (3) the CNS full analysis set (cFAS) group, which included patients for whom BICR detected measurable and non-measurable CNS metastases and patients with leptomeningeal metastases (LM).

A CNS measurable lesion was defined as a lesion that measured ≥ 10 mm in diameter by BICR. For patients with baseline CNS metastases, follow-up brain MRI and CT were performed every 6 weeks if possible. For patients with no evidence of a baseline CNS lesion, brain imaging was performed if clinically indicated.

### 2.3. Ethical Approval

The institutional review board approved ASTRIS at individual study centers (IRB No. YCC4-2018-1006, NCC2019-0083, CBU2019-02-007, KBSMC2019-02-009, CNUHH-2019-058). The trial was performed under the Good Clinical Practice guidelines and the Declaration of Helsinki. All included patients provided written informed consent before the enrollment of the trial.

### 2.4. Assessments and Statistical Analyses

The primary objective of this preplanned CNS analysis was intracranial progression-free survival (cPFS). Secondary objectives were intracranial objective response rate (cORR), intracranial disease control rate (cDCR), time to intracranial response (cTTR), duration of intracranial response (cDoR), and time to new CNS metastases (TTCM). The additional analysis included the assessment of leptomeningeal-response rate (LRR). All CNS efficacy was assessed according to Response Evaluation Criteria in Solid Tumors (RECIST), version 1.1, by BICR. cPFS was defined as the time from the start of the ASTRIS study until the date of objective intracranial disease progression or death (by any cause in the absence of progression), regardless of whether the subject withdraws from randomized therapy or receives another anti-cancer therapy prior to progression. cORR was defined as the percentage of patients who have at least one confirmed complete intracranial response (CR) or partial response (PR) prior to any evidence of progression (as defined by RECIST version 1.1).

Data obtained up until progression, or last evaluable assessment in the absence of progression, were included in the assessment of cORR. cDCR was defined as the proportion of patients with CR, confirmed PR, or stable disease (SD). cTTR was defined as the time from the start of the study until the first intracranial response. cDoR was defined as the time from the first observation of a CNS response until the first observation of intracranial progression or death. TTCM was defined as the time to the first detection of a new brain metastases or leptomeningeal metastases. For patients with LM, response evaluation was followed by RECIST 1.1 criteria as a non-target lesion. The data cutoff was 12 June 2019. Descriptive statistics were used for all variables and endpoints. Kaplan–Meier methods were used to calculate cPFS, cDCR, cTTR, cDoR, and TTCM.

## 3. Results

### 3.1. Patients

**Figure 1:**
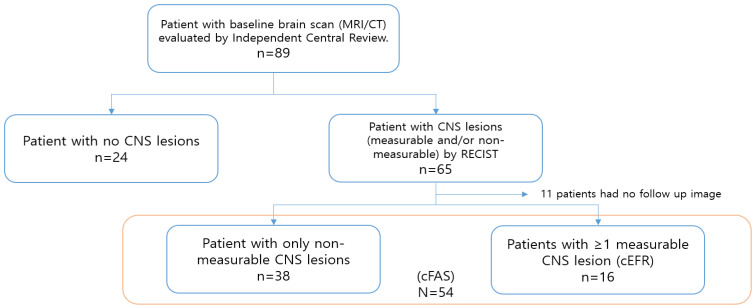
CONSORT diagram. cEFR, CNS evaluable for response set; cFAS, CNS full analysis set; CT, computed tomography; MRI, magnetic resonance imaging; RECIST, Response Evaluation Criteria in Solid Tumors.

Of 89 patients treated with osimertinib, the median age was 60, and most were female (69.7%). Seventy-seven patients met the Eastern Cooperative Oncology Group (ECOG) performance status (PS) criteria of 0–1, and all patients had adenocarcinoma histology harboring the EGFR T790M mutation (Table 1). Twenty-four patients did not have brain metastases at initial assessment, and eleven patients missed their regular imaging follow-up. Fifty-four patients were included in the cFAS group and sixteen patients in the cEFR group (Figure 1). Thirty-eight patients had prior brain radiotherapy, and nineteen patients showed LM disease on imaging (Table 1).

### 3.2. Osimertinib Efficacy

**Figure 2:**
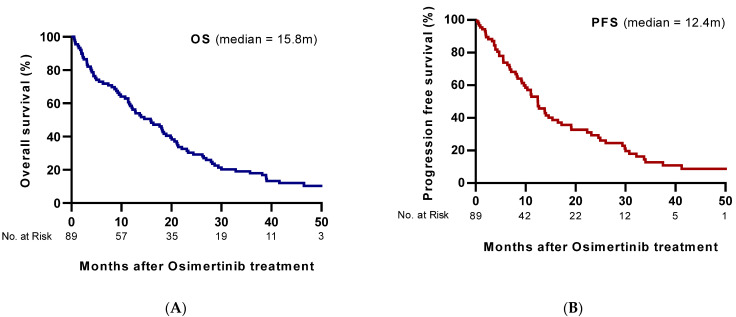
Kaplan–Meier estimates of total (**A**) overall survival and (**B**) progression-free survival.

For all patients, the median PFS and OS were 12.4 and 15.9 months respectively, indicating poor patient prognosis after progressing to osimertinib treatment (Figure 2). In the patients with at least one measurable lesion in the brain, cORR was 62.5% (95% CI, 38.3–82.6%). Zero (0%) and ten (63%) patients had a response of CR or PR, respectively. In the patients with any lesions in the brain, cORR was 38.9% (95% CI, 26.7–52.2%). Eleven (20.4%) and ten (18.5%) patients responded to CR or PR, respectively (Table 2).

For patients who received brain radiotherapy before osimertinib, cORR was 26.7% (cFAS, 95% CI, 13.5–44.1%) and 54.2% (95% CI, 34.7–72.7%) in patients with no prior brain radiotherapy (Table 3). In subgroups (with or without prior radiotherapy), cORR was significantly higher in patients without prior radiotherapy (*p* = 0.039). Regarding types of radiotherapy, 20 patients had whole brain radiotherapy and 10 patients had focal radiotherapy. Most patients with focal radiation progressed in non-irradiated fields (60%).

**Figure 3:**
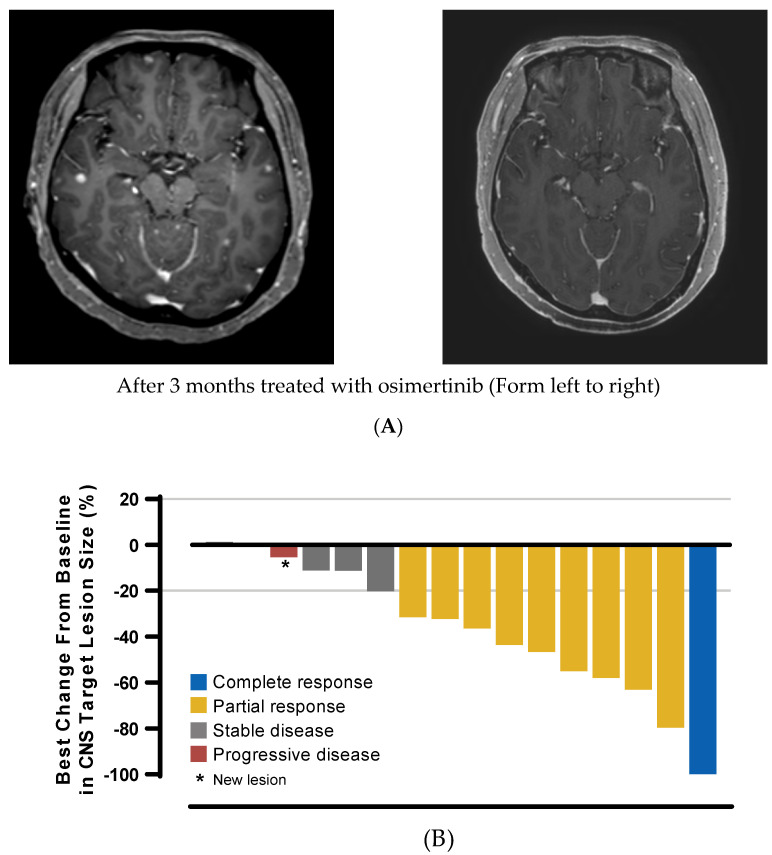
(**A**) Patient with LM disease who showed CR. (**B**) Best percentage change from baseline in target lesion size (CNS evaluable for response set). Best percentage change in target lesion size is the maximum reduction from baseline or the minimum increase from baseline in the absence of a reduction.

Of the 19 patients with LM, 2 patients had complete radiographic responses and 4 had partial radiographic responses. The median best percentage change after initial assessment of CNS target lesion size was −32.3% (range, −100% to +1.3%) (Figure 3). In the cFAS and cEFR groups, median cDoR was 12.1 months (95% CI, 8.5–15.8 months) and 7.6 months (95% CI, not calculable (NC)–5.7 months), respectively. Median weeks to response was 8.6 weeks for cFAS and 7.7 weeks for cEFR.

### 3.3. Concordance between CNS and Systemic Responses

In the cFAS, concordance between response (CR or PR) and nonresponse (SD or PD) categories for cORR and systemic ORR was 61.1% (33/54). The proportion of patients with systemic responses who also achieved a CNS response was 50% (15/30). Disagreements between CNS and the systemic responses were driven by assessment of SD in the CNS (including non-CR and non-PD), but the systemic assessment of PR (25.9%; 14/54), and disagreement on PD was 16.7% (9/54).

### 3.4. cPFS and Risk of CNS Progression

**Figure 4:**
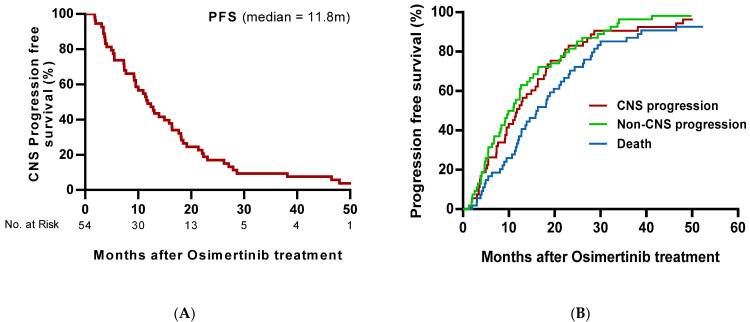
(**A**) Kaplan–Meier estimates of CNS progression-free survival (CNS full analysis set). (**B**) Cumulative incidence of CNS progression, taking into account competing risks of non-CNS progression and death by any cause (CNS full analysis set). The cumulative incidence function was calculated using a Fine and Gray model.

In the analyzable patients, the median cPFS was 11.8 months (95% CI, 8.7–14.8 months). Based on a Kaplan–Meier analysis, the estimated proportion of patients alive and CNS progression-free at 6 and 12 months was 72.2% (95% CI, 58.4–83.5%) and 48.2% (95% CI, 34.3–62.2%), respectively (Figure 4).

Based on competing risk analysis, the estimated probability of observing a CNS progression event at 3 months was 7.4% (95% CI, 2.1–17.9%), and 27.8% at 6 months (95% CI, 16.5–41.6%) (Figure 3). Two patients had new CNS metastasis, which occurred at 26.0 and 18.6 months, respectively.

## 4. Discussion

Osimertinib is the current standard treatment in patients with T790M-positive NSCLC, whose disease has progressed following first-line EGFR-TKI treatment. In this study, using the Korean CNS cohort (N = 466) of the ASTRIS study, we assessed the intracranial activity of osimertinib in 89 patients with EGFR T790M-positive advanced NSCLC with available brain scan at baseline.

This CNS analysis was preplanned, and neuroradiological BICR assessed CNS responses. The median cPFS of 11.8 months (95% CI, 8.7–14.8 months) and cORR of 38.9% in cFAS were consistent with previous reports [7]. In the cFAS, a high rate of agreement between CNS and systemic response to osimertinib was observed. Disagreement between CNS and the systemic response was primarily driven by assessment of SD in the CNS but the systemic assessment of PR, in which case, clinical management is the same. These results support the improvement in systemic response with osimertinib reported in the previous study population, including those patients with CNS metastases [6,10].

In the preclinical study, osimertinib showed greater BBB penetration compared to previous generation EGFR-TKIs and exhibited sustained metastatic brain tumor regression in mouse and nonhuman primate models [5]. The enhanced BBB penetrance of osimertinib compared to first- and second-generation EGFR-TKIs may explain the CNS responses observed in patients with advanced NSCLC. A pooled analysis of two prospective phase II trials resulted in a cORR of 54% and cDCR of 90% in cEFR [10]. In AURA3, cORR and cDCR were 70% and 93% in the osimertinib cEFR arm, respectively [6]. However, these previous studies are randomized controlled trials (RCTs) investigating the efficacy and safety of study drugs under well-designed, well-controlled, and standardized clinical conditions in a highly selected patient population; in these contexts, the outcomes from RCTs may not always reflect the real-world clinical practice. Patients may have severe comorbidities or poor performance status (e.g., ECOG PS ≥ 2), which are generally exclusion criteria in prospective clinical trials, or patients may have poor compliance, which is very different from protocol-specified patient care.

Thus, with increasing recognition, researchers and regulatory authorities are paying attention to the importance of real-world evidence and valuing the real-world data more than before [11]. ASTRIS was the largest global study to evaluate the safety and effectiveness of osimertinib in T790M-positive NSCLC in the real-world. The data regarding CNS activity were not available globally; however, our Korean CNS subset may further our understanding of the management of EGFR-mutant NSCLC brain metastases.

Our analysis adds to the evidence of CNS activity of osimertinib in T790M-positive NSCLC in the real-world by providing real-world experiences and broadening the study population. Additionally, though small, our study was a preplanned analysis, and CNS responses were assessed by BICR rather than investigator-assessed analysis, making our study more relevant.

Interpretation of CNS effects of osimertinib may be confounded by prior radiotherapy, and some studies have found that the BBB is more permeable to EGFR-TKI after radiotherapy, though others have not [12,13]. In the present analysis, CNS ORR with osimertinib was higher in patients who received no brain radiotherapy compared with patients who received prior radiotherapy. These results are inconsistent with AURA3 CNS analysis, in which the opposite effect was observed [6], likely due to the limited patient numbers in our study. Therefore, these data should be interpreted carefully. Regardless, osimertinib was observed to elicit CNS responses irrespective of prior brain radiotherapy in most studies.

Several limitations exist in our study. A major one is the small sample size of the subgroup analysis. Next, it was impossible to evaluate the CNS efficacy of osimertinib in patients without CNS metastases at the initial assessment. In addition, a limited number of patients were suspected to have LM only by imaging, with a lack of a cerebrospinal fluid (CSF) study. This may attribute to the invasiveness and technical difficulty of the procedure. Additionally, positive CSF cytology is known to be found on the initial lumbar puncture in only 50% of patients with LM, which makes physicians hesitate to perform the procedure for patients with bad performance status [14]. The role of osimertinib in treating patients with LM originating from NSCLC is being actively investigated, as these patients have a very poor prognosis with a median survival of 2–3 months [15]. The BLOOM study indicated that osimertinib is tolerable and exerts activity and manageable tolerability with 160 mg once daily [16]. Further real-world investigations into the efficacy of osimertinib treatment for LM patients are warranted.

## 5. Conclusions

In conclusion, the CNS outcomes in a Korean ASTRIS subset were consistent with those of previous prospective trials [6,7]. Our findings confirmed the consistent CNS bioavailability of osimertinib with fully matured OS and PFS for T790M-positive NSCLC with disease progression with first-line EGFR-TKI. An area of unmet need exists for better CNS penetration and efficacy.

## Figures and Tables

**Table 1:** Patient characteristics at initial assessment.

Patient Characteristics	
**Age**	**(years)**
Median (range)	60 (31–85)
**Sex**	***n* (%)**
Male	27 (30.3)
Female	62 (69.7)
**Histology**	
Adenocarcinoma	89 (100)
***EGFR* mutation**	
T790M	89 (100)
E19del	39 (43.8)
L858R	49 (55.1)
G719X	1 (1.1)
**ECOG PS score**	
0	36 (40.4)
1	41 (46.1)
2	21 (13.5)
**Prior brain therapy**	
Yes	38 (42.7)
No	27 (30.3)
No brain lesion	24 (27.0)
Baseline brain lesion *	
No metastasis	24 (27.0)
Brain metastasis only	46 (51.7)
Brain metastasis with LM	19 (21.3)

Abbreviations: EGFR, epidermal growth factor receptor; ECOG PS, Eastern Cooperative Oncology Group performance status; LM, leptomeningeal metastasis. * All the patients were evaluated with MRI.

**Table 2:** CNS response to osimertinib.

Analysis Set/Response	cFAS (*N* = 54) *n* (%)(95% CI)	cEFR (*N* = 16) *n* (%) (95% CI)
CNS ORR, *n* (%) (95% CI)	21 (38.9) (26.7–52.2)	10 (62.5) (38.3–82.6)
Complete response (CR)	11 (20.4)	0 (0)
Partial response (PR)	10 (18.5)	10 (62.5)
Stable disease (SD)	5 (9.3)	5 (31.3)
Progressive disease	2 (3.7)	1 (6.2)
non-CR, non-PD ^†^	26 (48.1)	-
CNS DCR *n* (%) (95% CI)	52 (96.3) (88.6–99.2)	15 (93.8) (74.3–99.3)
CNS DoR ^§^		
Median, months (95% CI)	12.1 (8.5–15.8)	7.6 (NC–17.8)
Time to onset of response		
Median, weeks (interquartile range)	8.6 (7.7–26.0)	7.7 (7.0–8.6)
≤9 weeks, No. (%)	12 (57.1)	8 (80.0)

Abbreviations: cEFR, CNS evaluable for response set; cFAS, CNS full analysis set; CR, complete response; DCR, disease control rate; DoR, duration of response; NC, not calculable; ORR, objective response rate; PD, progressive disease; PR, partial response. ^†^ For patients with non-measurable lesions only, response could be classed as CR, non-CR, non-PD, or PD. ^§^ Duration of response is time from first documentation of CR/PR until date of progression or death in the absence of progression.

**Table 3:** CNS objective response rate in the cFAS by prior brain radiotherapy.

Treatment	Patients with a Response * n (%) (95% CI)	*p*
Prior radiotherapy before randomization (*n* = 30) **	8 (26.7) (13.5–44.1)	0.039
No prior radiotherapy before randomization (*n* = 24)	13 (54.2) (34.7–72.7)	

Abbreviation: cFAS, CNS full analysis set. * Patients with non-measurable disease only are included. ** Median interval from end of radiotherapy to CNS imaging was 5.95 months.

## References

[1] Peters S., Bexelius C., Munk V., Leighl N. (2016). The impact of brain metastasis on quality of life, resource utilization and survival in patients with non-small-cell lung cancer. Cancer Treat. Rev..

[2] Heon S., Yeap B.Y., Lindeman N.I., Joshi V.A., Butaney M., Britt G.J., Costa D.B., Rabin M.S., Jackman D.M., Johnson B.E. (2012). The impact of initial gefitinib or erlotinib versus chemotherapy on central nervous system progression in advanced non-small cell lung cancer with EGFR mutations. Clin. Cancer Res..

[3] Camidge D.R., Pao W., Sequist L.V. (2014). Acquired resistance to TKIs in solid tumours: Learning from lung cancer. Nat. Rev. Clin. Oncol..

[4] Rangachari D., Yamaguchi N., VanderLaan P.A., Folch E., Mahadevan A., Floyd S.R., Uhlmann E.J., Wong E.T., Dahlberg S.E., Huberman M.S. (2015). Brain metastases in patients with EGFR-mutated or ALK-rearranged non-small-cell lung cancers. Lung Cancer.

[5] Ballard P., Yates J.W., Yang Z., Kim D.W., Yang J.C., Cantarini M., Pickup K., Jordan A., Hickey M., Grist M. (2016). Preclinical Comparison of Osimertinib with Other EGFR-TKIs in EGFR-Mutant NSCLC Brain Metastases Models, and Early Evidence of Clinical Brain Metastases Activity. Clin. Cancer Res..

[6] Wu Y.L., Ahn M.J., Garassino M.C., Han J.Y., Katakami N., Kim H.R., Hodge R., Kaur P., Brown A.P., Ghiorghiu D. (2018). CNS Efficacy of Osimertinib in Patients With T790M-Positive Advanced Non-Small-Cell Lung Cancer: Data from a Randomized Phase III Trial (AURA3). J. Clin. Oncol..

[7] Reungwetwattana T., Nakagawa K., Cho B.C., Cobo M., Cho E.K., Bertolini A., Bohnet S., Zhou C., Lee K.H., Nogami N. (2018). CNS Response to Osimertinib Versus Standard Epidermal Growth Factor Receptor Tyrosine Kinase Inhibitors in Patients With Untreated EGFR-Mutated Advanced Non-Small-Cell Lung Cancer. J. Clin. Oncol..

[8] Cho B.C., Kim D.W., Park K., Lee J.S., Yoo S.S., Kang J.H., Lee S.Y., Kim C.H., Jang S.H., Kim Y.C. (2020). Real-world use of osimertinib in non-small cell lung cancer: ASTRIS study Korean subgroup analysis. Curr. Med. Res. Opin..

[9] Marinis F., Wu Y.L., de Castro G., Chang G.C., Chen Y.M., Cho B.C., Freitas H.C., Jiang L., Kim S.W., Martin C. (2019). ASTRIS: A global real-world study of osimertinib in >3000 patients with EGFR T790M positive non-small-cell lung cancer. Future Oncol..

[10] Goss G., Tsai C.M., Shepherd F.A., Ahn M.J., Bazhenova L., Crinò L., de Marinis F., Felip E., Morabito A., Hodge R. (2018). CNS response to osimertinib in patients with T790M-positive advanced NSCLC: Pooled data from two phase II trials. Ann. Oncol..

[11] Khozin S., Blumenthal G.M., Pazdur R. (2017). Real-world Data for Clinical Evidence Generation in Oncology. J. Natl. Cancer Inst..

[12] Zeng Y.-D., Liao H., Qin T., Zhang L., Wei W.-D., Liang J.-Z., Xu F., Dinglin X.-X., Ma S.-X., Chen L.-K. (2015). Blood-brain barrier permeability of gefitinib in patients with brain metastases from non-small-cell lung cancer before and during whole brain radiation therapy. Oncotarget.

[13] Fang L., Sun X., Song Y., Zhang Y., Li F., Xu Y., Ma S., Lin N. (2015). Whole-brain radiation fails to boost intracerebral gefitinib concentration in patients with brain metastatic non-small cell lung cancer: A self-controlled, pilot study. Cancer Chemother. Pharmacol..

[14] Grossman S.A., Krabak M.J. (1999). Leptomeningeal carcinomatosis. Cancer Treat. Rev..

[15] Chamberlain M., Soffietti R., Raizer J., Ruda R., Brandsma D., Boogerd W., Taillibert S., Groves M.D., Le Rhun E., Junck L. (2014). Leptomeningeal metastasis: A Response Assessment in Neuro-Oncology critical review of endpoints and response criteria of published randomized clinical trials. Neuro Oncol.

[16] Yang J.C.H., Kim S.-W., Kim D.-W., Lee J.-S., Cho B.C., Ahn J.-S., Lee D.H., Kim T.M., Goldman J.W., Natale R.B. (2020). Osimertinib in Patients With Epidermal Growth Factor Receptor Mutation–Positive Non–Small-Cell Lung Cancer and Leptomeningeal Metastases: The BLOOM Study. J. Clin. Oncol..

